# Deletion of the Major Facilitator Superfamily Transporter *fptB* Alters Host Cell Interactions and Attenuates Virulence of Type A Francisella tularensis

**DOI:** 10.1128/IAI.00832-17

**Published:** 2018-02-20

**Authors:** Phillip M. Balzano, Aimee L. Cunningham, Christen Grassel, Eileen M. Barry

**Affiliations:** a Institute for Global Health, Center for Vaccine Development, University of Maryland School of Medicine, Baltimore, Maryland, USA; The University of Texas at Austin

**Keywords:** FPT mutants, *Francisella tularensis*, major facilitator superfamily, amino acid transport, tularemia

## Abstract

Francisella tularensis is a Gram-negative, facultative, intracellular coccobacillus that can infect a wide variety of hosts. In humans, F. tularensis causes the zoonosis tularemia following insect bites, ingestion, inhalation, and the handling of infected animals. The fact that a very small inoculum delivered by the aerosol route can cause severe disease, coupled with the possibility of its use as an aerosolized bioweapon, has led to the classification of Francisella tularensis as a category A select agent and has renewed interest in the formulation of a vaccine. To this end, we engineered a type A strain SchuS4 derivative containing a targeted deletion of the major facilitator superfamily (MFS) transporter *fptB*. Based on the attenuating capacity of this deletion in the F. tularensis LVS background, we hypothesized that the deletion of this transporter would alter the intracellular replication and cytokine induction of the type A strain and attenuate virulence in the stringent C57BL/6J mouse model. Here we demonstrate that the deletion of *fptB* significantly alters the intracellular life cycle of F. tularensis, attenuating intracellular replication in both cell line-derived and primary macrophages and inducing a novel cytosolic escape delay. Additionally, we observed prominent differences in the *in vitro* cytokine profiles in human macrophage-like cells. The mutant was highly attenuated in the C57BL/6J mouse model and provided partial protection against virulent type A F. tularensis challenge. These results indicate a fundamental necessity for this nutrient transporter in the timely progression of F. tularensis through its replication cycle and in pathogenesis.

## INTRODUCTION

Francisella tularensis is a Gram-negative, facultative, intracellular coccobacillus that is the causative agent of the zoonosis tularemia (1, 2). Two subspecies of Francisella, F. tularensis subsp. tularensis (type A) and F. tularensis subsp. holarctica (type B), account for virtually all instances of disease in humans, although a third, F. tularensis subsp. novicida, is not infectious in humans but is often used as a laboratory model. While there is no documented case of human-to-human transmission, F. tularensis can be spread to humans via the inhalation of infected aerosols, the bite of insect vectors, the ingestion of contaminated food or water, and the handling of infected animals (3). Cutaneous exposure, most often via an insect vector, accounts for much of the disease burden and can lead to a protracted although rarely fatal infection (4, 5). Conversely, the inhalation of as few as 10 to 15 bacteria of the virulent type A strain can cause serious illness in humans, with a mortality rate approaching 50% if left untreated (6, 7). Several countries had utilized Francisella as a biological weapon during the middle of the 20th century due to its high virulence, low inoculum, and ease of delivery by the aerosol route (8). For this reason, the CDC has designated Francisella tularensis subsp. tularensis a tier 1 category A select agent and a high priority for countermeasure development.

Currently, there is no licensed vaccine against F. tularensis, but development efforts continue in light of the potential threat to public safety. Past development efforts included component and killed whole-cell, or “Foshay,” vaccines, but these vaccines were met with limited success (9–12). To date, the live-attenuated vaccine strategy has shown the most promise. One candidate strain derived from F. tularensis subsp. holarctica by the Soviet Union through the 1940s and 1950s was transferred to the United States in 1956 and further passaged and became known as “live vaccine strain” (LVS) (7). F. tularensis LVS was tested extensively in clinical trials and demonstrated the feasibility of the live-vaccine approach in conferring at least partial protection against type A challenge. Subsequently, multiple attenuated live vaccine candidates have been engineered by utilizing the F. tularensis subsp. novicida, F. tularensis LVS, and F. tularensis type A backgrounds (6, 13–22). These new vaccine candidates have shown various degrees of efficacy in animal models, and some are being further developed for use in humans. An additional outcome of these studies has been the discovery of novel information about the pathogenic process of F. tularensis, including alterations to and impacts on the pathogenic life cycle incurred by the targeted deletion of various genes in the attenuation process.

A hallmark of Francisella infection is the ability to induce phagocytosis via several routes to gain entry into many cell types, especially macrophages, where it rapidly escapes the phagosome to replicate to high numbers in the cytosol (23–27). Because of the distinctive tetra-acylated structure of its lipopolysaccharide lipid A component, Francisella is largely able to evade Toll-like receptor 4 (TLR4)-mediated recognition (28). Rather, Francisella initiates signaling through TLR2, resulting in the production of proinflammatory cytokines (29, 30), and, in a manner dependent on guanylate binding proteins (GBPs), STING, and mitochondrial reactive oxygen species, activates the AIM2 inflammasome (31–34). The virulence of Francisella stems from its ability to multiply to high numbers in the cytosols of infected cells, especially macrophages (35). As a result, many attenuation attempts have targeted metabolic or nutrient acquisition genes (13, 15–17, 36–38). We demonstrated previously that deletions in three of eight Francisella phagosomal transporter (*fpt*) genes, encoding members of the major facilitator superfamily (MFS) of secondary transporters, in the F. tularensis LVS background resulted in significant *in vitro* and *in vivo* phenotypes: the intracellular replication of these strains in macrophages and hepatocytes was reduced, and the mutants were attenuated in BALB/c mice. Furthermore, immunization of mice with F. tularensis strain LVSΔ*fptB*, LVSΔ*fptE*, or LVSΔ*fptG* protected mice against homologous lethal challenge (13). We hypothesized that engineering deletion mutations in one of the most promising of these genes, *fptB*, an isoleucine transporter (38), in the F. tularensis type A background would result in a protective vaccine candidate strain. Here we report that an F. tularensis strain SchuS4 mutant lacking *fptB* demonstrates altered intracellular replication and cytokine release kinetic profiles in macrophages and is attenuated in mice, indicating its essential role in the intracellular life cycle and virulence of F. tularensis.

## RESULTS

### Generation of type A Francisella tularensis SchuS4 strains with an *fpt* deletion.

**FIG 1:**
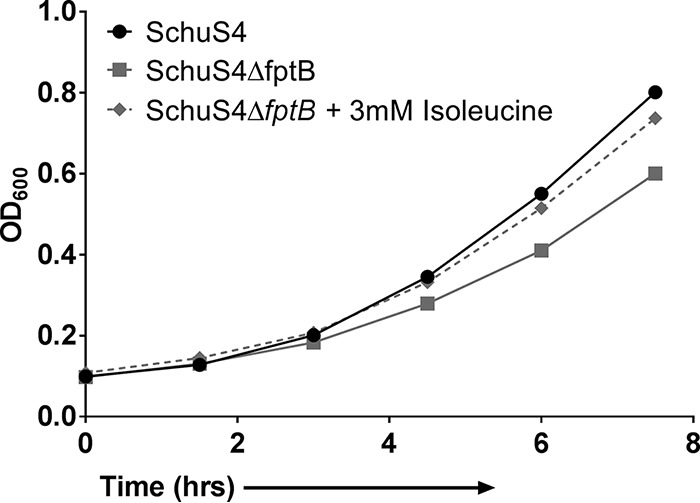
Growth in Chamberlain's defined medium. Growth kinetics of WT F. tularensis SchuS4 and SchuS4Δ*fptB* in Chamberlain's defined medium with or without supplementation with 3 mM isoleucine were examined. Data are from a single representative experiment (*n* = 3).

An F. tularensis type A strain with an unmarked deletion of *fptB* was constructed by using allelic exchange technology as described previously (13). The deletion of the target gene and the generation of F. tularensis mutant strain SchuS4Δ*fptB* were confirmed by PCR using primers originating in both gene flanks and intragenically (see Fig. S1 in the supplemental material). The substrate for *fptB* has been reported to be isoleucine in both F. tularensis subsp. holarctica and F. tularensis subsp. novicida (38). Given the 99% identity between the type A and type B gene sequences, we first confirmed that isoleucine was the substrate for *fptB* using Chamberlain's defined medium (CDM); the growth of F. tularensis SchuS4Δ*fptB* was reduced compared to that of the wild type (WT) in CDM but could be restored to WT levels by the addition of exogenous isoleucine (Fig. 1).

### Mutant strain SchuS4Δ*fptB* is deficient in intracellular replication and cellular escape in macrophages.

**FIG 2:**
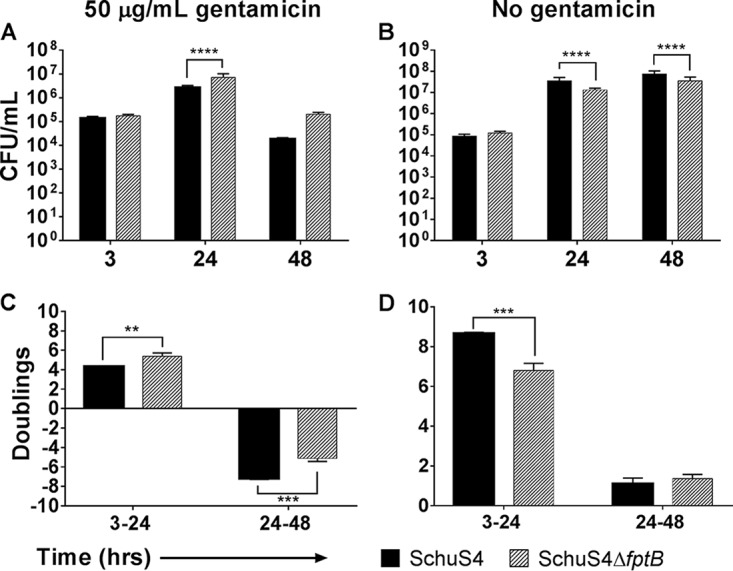
F. tularensis SchuS4Δ*fptB* exhibits altered intracellular replication kinetics in THP-1 cells. THP-1 cells were infected with the F. tularensis WT strain or SchuS4Δ*fptB* at an MOI of 100 for 2 h. Cells were then washed twice with PBS and incubated with 50 μg/ml gentamicin either for 1 h followed by washing and incubation without antibiotics (B and D) or for the duration of the experiment (A and C). Intracellular bacteria were enumerated at 3, 24, and 48 hpi, and the number of doublings was calculated between each interval. Data are presented as means ± standard errors of the means with three biological replicates for each experiment. Data are from a single representative experiment (*n* = 5). **, *P* < 0.01; ***, *P* < 0.001; ***, *P* < 0.001; ****, *P* < 0.0001 (average CFU were analyzed by two-way ANOVA, and calculated doublings were analyzed by a two-sided *t* test with Benjamini, Krieger, and Yekutieli *P* value adjustment).

The virulence of Francisella is dependent, in part, on its ability to replicate to high numbers intracellularly, especially in macrophages (35). The replication kinetics of the *fpt* mutant strain was compared to that of WT F. tularensis SchuS4 initially in THP-1 human macrophage-like cells and HepG2 human hepatic carcinoma cells. In order to observe only the initial round of invasion and replication, a bactericidal concentration of gentamicin was maintained in the culture medium to prevent cell-to-cell spread. Under these conditions, there were significantly higher numbers of the mutant strain in THP-1 macrophages at 24 h postinfection (hpi) than of the WT strain (*P* < 0.0001) (Fig. 2A). WT F. tularensis doubled 3 to 4.5 times in the first 24 h, compared to 5 to 5.4 times for F. tularensis SchuS4Δ*fptB*, significantly more than the WT strain (*P* < 0.01) (Fig. 2C).

**FIG 3:**
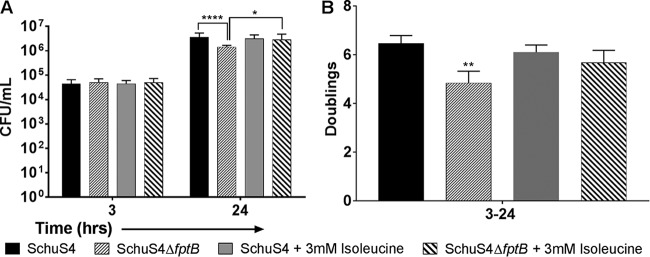
Excess isoleucine restores F. tularensis SchuS4Δ*fptB* replication kinetics to WT levels in THP-1 cells. THP-1 cells were infected at an MOI of 100 for 2 h. Cells were then washed twice with PBS and incubated with 50 μg/ml gentamicin for 1 h before being returned to medium lacking gentamicin. Intracellular bacteria were enumerated at 3, 24, and 48 h postinfection. Data are presented as means ± standard errors of the means with three biological replicates for each experiment. Data are from a single representative experiment (*n* = 2). *, *P* < 0.05; **, *P* < 0.01; ****, *P* < 0.0001 (average CFU were analyzed by two-way ANOVA, and calculated doublings were analyzed by a two-sided *t* test with Benjamini, Krieger, and Yekutieli *P* value adjustment).

When cell-to-cell spread was allowed to proceed by the removal of gentamicin from the medium postinvasion, F. tularensis SchuS4Δ*fptB* exhibited a significant (*P* < 0.0001) replication defect as evidenced by reduced numbers of intracellular bacteria and significantly fewer doublings (*P* < 0.001) through 24 h (Fig. 2B and D). Without gentamicin, F. tularensis SchuS4Δ*fptB* doubled only 6.8 times, compared to 8.7 times for the WT (Fig. 2D). These data suggest that F. tularensis SchuS4Δ*fptB* has a reduced intracellular replication rate compared to that of the WT and is delayed in cellular escape. *trans* complementation of the *fptB* gene with an intact plasmid-borne gene copy restored WT replication kinetics for the mutant strain in THP-1 cells (see Fig. S2 in the supplemental material). Furthermore, the addition of 3 mM isoleucine, the reported substrate for FptB (38), to the culture medium of THP-1 cells infected with F. tularensis SchuS4Δ*fptB* was able to rescue the replication defect in a fashion similar to that in broth assays. The addition of exogenous isoleucine resulted in equivalent numbers of intracellular bacteria for F. tularensis SchuS4Δ*fptB* and the WT at 24 hpi (Fig. 3A). Similarly, the calculated doubling rate for the mutant in the presence of isoleucine was significantly higher than that of the mutant without isoleucine (*P* < 0.05) and statistically equivalent to that of the WT (Fig. 3B).

Finally, intracellular growth kinetics were tested in the human hepatic carcinoma cell line HepG2. Previous work demonstrated that the LVSΔ*fptB* mutant exhibited a replication defect compared to the parental LVS strain, with fewer mutant bacteria being present intracellularly at 24 and 48 hpi. In contrast, SchuS4Δ*fptB* did not exhibit a growth defect or delayed cell escape in this cell type (data not shown).

### F. tularensis SchuS4Δ*fptB* induces delayed cell death in THP-1 cells.

**FIG 4:**
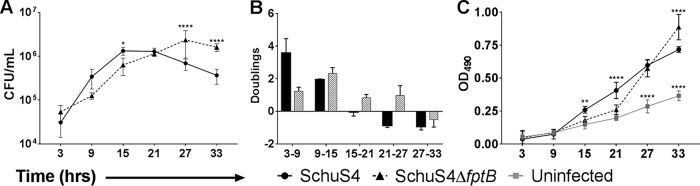
F. tularensis SchuS4Δ*fptB* induces a delay in cell death and LDH release. THP-1 cells were infected with the F. tularensis WT strain or SchuS4Δ*fptB* at an MOI of 100 for 2 h. Cells were then washed twice with PBS and incubated with 50 μg/ml gentamicin for the duration of the experiment. Intracellular bacteria were enumerated at 3, 9, 15, 21, 27, and 33 hpi; doublings were calculated between each time interval; and supernatants were analyzed for LDH release. Data are presented as means ± standard errors of the means with three biological replicates for each experiment. Data are from a single representative experiment (*n* = 4). *, *P* < 0.05; **, *P* < 0.01; ****, *P* < 0.0001 (average CFU were analyzed by two-way ANOVA, and calculated doublings were analyzed by a two-sided *t* test with Benjamini, Krieger, and Yekutieli *P* value adjustment).

To examine more closely the escape delay phenotype displayed by F. tularensis SchuS4Δ*fptB*, invasion assays with THP-1 macrophages were carried out under cell spread-limiting conditions with time points every 6 h during 33 h of infection. Positive doubling rates were interpreted as an indication that replication was outpacing escape, and negative doubling rates were interpreted as an indication that escape was outpacing replication. Supernatants were assayed for the release of lactate dehydrogenase (LDH), an intracellular enzyme whose release is indicative of cell death (39, 40). Supporting our previous findings with THP-1 cells, F. tularensis SchuS4Δ*fptB* exhibited a significant growth defect that was quantified as reduced intracellular bacterial numbers, evident by 15 hpi (*P* < 0.05) (Fig. 4A), and reduced doublings at between 3 and 9 hpi (Fig. 4B). F. tularensis SchuS4Δ*fptB* continued to double for 12 h beyond that of the WT, exhibiting increasing numbers of CFU (Fig. 4A) and positive doubling values (Fig. 4B) through 27 hpi, 12 h beyond that of the WT. In fact, F. tularensis SchuS4Δ*fptB* reached significantly higher peak intracellular numbers than did the WT (*P* < 0.0001). The release of LDH from F. tularensis SchuS4Δ*fptB*-infected cells was similarly and significantly (*P* < 0.0001) delayed compared to that from WT-infected cells (Fig. 4C). The release kinetics indicate a 12-h delay in the onset of cell death.

**FIG 5:**
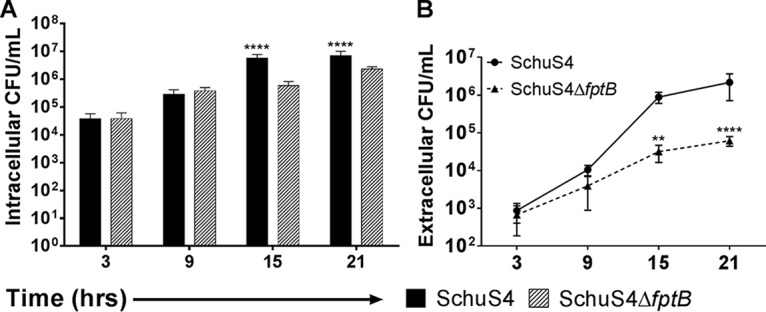
F. tularensis SchuS4Δ*fptB* is delayed in release to infected cell supernatants. THP-1 cells were infected with the F. tularensis WT strain or SchuS4Δ*fptB* at an MOI of 100 for 2 h. Cells were then washed twice with PBS and incubated with 50 μg/ml gentamicin for 1 h before being washed twice more with PBS and being returned to medium lacking gentamicin. Intracellular and extracellular bacteria were enumerated at 3, 9, 15, and 21 hpi. Data are presented as means ± standard errors of the means with three biological replicates for each experiment. Data are from a single representative experiment (*n* = 2). **, *P* < 0.01; ****, *P* < 0.0001 (average CFU were analyzed by two-way ANOVA, and calculated doublings were analyzed by a two-sided *t* test with Benjamini, Krieger, and Yekutieli *P* value adjustment).

To further assess the differences in cellular escape between F. tularensis SchuS4Δ*fptB* and the WT, bacteria were sampled from the supernatants of infected THP-1 cells cultured without antibiotics as well as from the intracellular compartment. As described above, there were significantly fewer F. tularensis SchuS4Δ*fptB* than WT CFU by 15 hpi (*P* < 0.0001) (Fig. 5A). By 15 hpi, significantly more WT F. tularensis bacteria were found in the supernatants of infected cells (*P* < 0.01) than SchuS4Δ*fptB* bacteria (Fig. 5B). This increase in the number of extracellular WT F. tularensis bacteria correlates with the significant increase in LDH release seen previously at the 15-hpi time point (Fig. 4C). These results support a role for *fptB* in intracellular replication and timely escape from the cell.

### F. tularensis SchuS4Δ*fptB* elicits a delayed early proinflammatory response in host cells.

**FIG 6:**
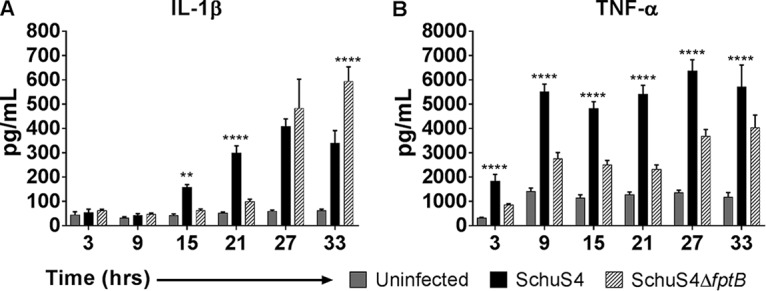
F. tularensis SchuS4Δ*fptB* elicits altered inflammatory responses. THP-1 cells were infected at an MOI of 100 for 2 h. Cells were then washed twice with PBS and incubated with 50 μg/ml gentamicin for the duration of the experiment. Cell-free supernatants were collected at 3, 9, 15, 21, 27, and 33 hpi, and cytokine contents were measured by using sandwich ELISAs. Data are presented as means ± standard errors of the means with three biological replicates for each experiment. Data are from a single representative experiment (*n* = 5). **, *P* < 0.01; ****, *P* < 0.0001 (by two-way ANOVA).

The production of TLR2-dependent cytokines and the activation of STING and the AIM2 inflammasome are necessary for the initiation of the host cytokine response to F. tularensis infection (30, 41–43). The altered intracellular replication kinetics of F. tularensis SchuS4Δ*fptB* in macrophages suggested that infection with the mutant strain would likely result in modified interactions with innate immune components. The levels of secretion of two important early proinflammatory cytokines, tumor necrosis factor alpha (TNF-α) and interleukin-1β (IL-1β), were measured in supernatants from the infected THP-1 samples from the above-described assay. The release of IL-1β was delayed in F. tularensis SchuS4Δ*fptB*-infected cells until 27 hpi, 12 h later than when WT-infected cells began secreting IL-1β at a rate above that of uninfected cells (Fig. 6A). At 27 hpi, IL-1β secretion from cells infected with F. tularensis SchuS4Δ*fptB* reached levels equivalent to that of the WT. The timing of IL-1β release for both the mutant and the WT coincides with that of LDH release, demonstrating a correlation between cell death and mature IL-1β release. Similarly, infection of THP-1 cells with WT F. tularensis induced a rapid and robust induction of TNF-α. In contrast, F. tularensis SchuS4Δ*fptB* induced the release of significantly less TNF-α than in WT-infected cells at all time points measured (*P* < 0.0001), despite increasing numbers of intracellular bacteria (Fig. 6B).

### F. tularensis SchuS4Δ*fptB* is replication deficient in primary macrophages.

**FIG 7:**
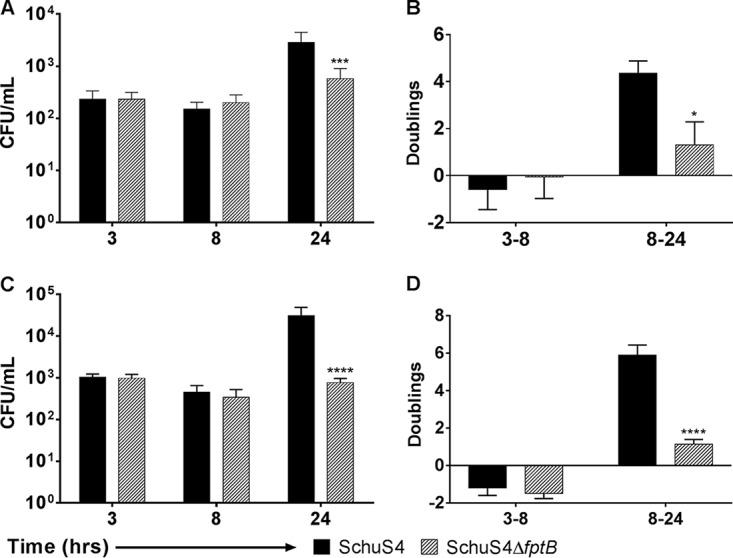
F. tularensis SchuS4Δ*fptB* is deficient in replication in primary cells. Human MDM cells (A and B) and mouse BMDM cells (C and D) were infected at an MOI of 10 for 2 h. Cells were then washed twice with PBS and incubated with 50 μg/ml gentamicin for 1 h before being returned to medium lacking gentamicin. Intracellular bacteria were enumerated at 3, 8, and 24 h postinfection. Data are presented as means ± standard errors of the means with three biological replicates for each experiment. Data are from a single representative experiment (*n* = 4). *, *P* < 0.05; ***, *P* < 0.001; ****, *P* < 0.0001 (average CFU were analyzed by two-way ANOVA, and calculated doublings were analyzed by a two-sided *t* test with Benjamini, Krieger, and Yekutieli *P* value adjustment).

In order to bridge studies between the THP-1 cell line and *in vivo* experiments in mice, the replication kinetics of the *fpt* mutant strains was also examined in human monocyte-derived macrophages (hMDMs) and bone marrow-derived macrophages (BMDMs) from C57BL/6J mice. We hypothesized that growth attenuation in primary cells would be predictive of attenuation *in vivo*. Significantly fewer intracellular F. tularensis SchuS4Δ*fptB* mutant bacteria (*P* < 0.001) than WT bacteria were counted at 24 hpi (Fig. 7A) in hMDMs. Correspondingly, the number of doublings for F. tularensis SchuS4Δ*fptB* was significantly lower than that for the WT at between 8 and 24 hpi (1.5 versus 4.2 doublings, respectively; *P* < 0.05) (Fig. 7B). Similar trends were observed in mouse BMDMs. F. tularensis SchuS4Δ*fptB* elicited significantly fewer intracellular CFU at 24 hpi (Fig. 7C). This corresponded to a significant reduction in the calculated doubling rate between F. tularensis SchuS4Δ*fptB* and the WT at between 8 and 24 hpi (1.2 versus 6.1 doublings, respectively; *P* < 0.0001) (Fig. 7D).

### SchuS4Δ*fptB* is highly attenuated in the C57BL/6J mouse model.

The altered intracellular replication kinetics and cytokine induction patterns suggested a role for *fptB* in Francisella virulence. The C57BL/6J mouse model was used to assess pathogenic defects of these mutant strains, as WT type A F. tularensis has an intranasal 50% lethal dose (LD_50_) of <10 CFU in this model (44). Groups of 4 mice were inoculated intranasally with 5 × 10^1^ CFU of the WT or 5 × 10^1^ to >7 × 10^6^ CFU of the mutant strains. WT-inoculated mice steadily lost weight over the course of 5 days before succumbing to infection on day 5, exhibiting overt signs of sickness beginning late on day 4 or early on day 5. In contrast, no mice inoculated with F. tularensis SchuS4Δ*fptB* up to the highest dose succumbed to infection (Table 1). No weight loss or clinical signs of sickness were evident in any F. tularensis SchuS4Δ*fptB*-inoculated mice, demonstrating attenuation and an LD_50_ of >7.6 × 10^6^ CFU.

**TABLE 1:** Attenuation of the *fptB* deletion strain in C57BL/6J mice

Strain	Intranasal dose (CFU)	No. of surviving mice/total no. of mice
SchuS4	1.10 × 10^1^	0/2
SchuS4	1.90 × 10^1^	0/4
SchuS4	4.30 × 10^1^	0/4
SchuS4	5.30 × 10^1^	0/4
SchuS4Δ*fptB*	5.40 × 10^1^	3/3
SchuS4Δ*fptB*	3.10 × 10^2^	4/4
SchuS4Δ*fptB*	5.60 × 10^2^	3/3
SchuS4Δ*fptB*	1.01 × 10^3^	4/4
SchuS4Δ*fptB*	8.70 × 10^3^	4/4
SchuS4Δ*fptB*	9.80 × 10^4^	4/4
SchuS4Δ*fptB*	1.02 × 10^5^	2/2
SchuS4Δ*fptB*	1.06 × 10^6^	4/4
SchuS4Δ*fptB*	7.60 × 10^6^	3/3

### F. tularensis SchuS4Δ*fptB* vaccination protects mice against virulent F. tularensis SchuS4 challenge.

Given the high level of attenuation of SchuS4Δ*fptB*, we assessed the ability of this strain to protect mice against lethal challenge. Groups of 4 mice in two independent experiments were inoculated with a single dose of ∼1 × 10^6^ CFU of F. tularensis SchuS4Δ*fptB* before subsequent WT challenge 28 days later with doses ranging from 7 CFU to 360 CFU. Ten of 26 vaccinated mice from two separate experiments survived challenge, as did 1 unvaccinated control, resulting in an efficacy value of 28% (Table 2). An increased delay in the time to death was observed in a challenge-dose-dependent manner (Table 2). All protected mice showed no overt signs of illness, with little to no loss of weight following challenge. These data suggest that F. tularensis SchuS4Δ*fptB* has a limited protective capacity but demonstrates the first example of protection by an MFS transporter-targeted vaccine strain from type A challenge.

**TABLE 2:** Protection against wild-type challenge after vaccination with SchuS4Δ*fptB*

Vaccine inoculum (CFU)	Challenge dose of SchuS4 (CFU)	No. of surviving mice/total no. of mice	Delays in time to death of individual mice (days)	Avg delay in time to death (days)
					7.30 × 10^5^	7	2/5	8, 9, 8, >14, >14	4
7.30 × 10^5^	70	1/4	7, 6, 6, >14	2
7.30 × 10^5^	162	2/5	6, 6, 6, >14, >14	1.67
PBS	350	0/3	4, 4, 5	
1.14 × 10^6^	39	2/4	7, 10, >14, >14	3.5
1.14 × 10^6^	78	1/4	6, 7, 9, >14	2.33
1.14 × 10^6^	360	2/4	7, 6, >14, >14	1.5
PBS	39	1/4	5, 5, 5, >14	

## DISCUSSION

The formulation of a licensed, efficacious, live-attenuated vaccine against Francisella tularensis has remained an elusive goal despite increased efforts since the anthrax bioterror attacks of 2001. Previous studies in humans with F. tularensis LVS in the 1960s demonstrated the proof of concept for the protective capacity of the live-attenuated vaccine strategy and pointed the way forward for the engineering of live-attenuated strains harboring targeted deletions (6, 13, 14, 19, 45). Here we investigated the potential of one MFS transporter, FptB, as an attenuating target in the virulent type A strain and the effects of its loss on the intracellular life cycle of type A F. tularensis. Our previous studies demonstrated the importance of the *fptB* gene in the F. tularensis LVS background for both *in vitro* replication kinetics and *in vivo* virulence (13). F. tularensis LVSΔ*fptB* exhibited a severe replication defect in multiple cell types, and the strain was both attenuated and protective in the BALB/c model (13). We hypothesized that given the high protein identity between the F. tularensis LVS and type A strains for this protein (99% for FptB), similar phenotypes would be observed in the clinically relevant type A background.

F. tularensis SchuS4Δ*fptB* displayed a significant growth defect in macrophages, both cell line derived and primary, similar to observations with the F. tularensis LVS-derived mutant, but, in contrast to our previous findings with LVS, did not harbor a replication defect in HepG2 cells (13, 38). The restriction of intracellular growth defects to macrophages and not hepatic cells may stem from the fact that the substrate of FptB, isoleucine, is regulated in macrophages. Isoleucine is one of nine amino acids that are depleted by activated macrophages (46). This, coupled with the fact that excess isoleucine in culture rescues the growth of F. tularensis SchuS4Δ*fptB*, implies that isoleucine is a rate-limiting nutrient for the intracellular replication of Francisella tularensis and that there is likely another, previously hypothesized (38), lower-affinity transporter that contributes to the transport of isoleucine in the absence of FptB. It may be that FptB functions as a high-affinity transporter to scavenge isoleucine intracellularly, even at depleted levels.

In addition to an intracellular replication defect, these data support an escape delay for SchuS4Δ*fptB*. Multiple studies have demonstrated that F. tularensis does not replicate until it escapes from the phagosome (47–49). The positive intracellular replication rate of SchuS4Δ*fptB*, measured as early as 3 to 9 hpi, demonstrates that the mutant strain has escaped from the phagosome, similarly to the WT, and is replicating in the cytosol albeit at a reduced rate. However, the mutant continues to replicate intracellularly for an additional 12 h beyond when WT F. tularensis is primarily escaping. Further evidence demonstrating a delay in the emergence of F. tularensis SchuS4Δ*fptB* in infected cell supernatants compared to the WT, and a corresponding delay in the onset of LDH release from mutant-infected cells compared to WT-infected cells, supports a delayed-cellular-escape phenotype. Specifically, at 21 hpi, F. tularensis SchuS4Δ*fptB* and the WT strain are present at similar intracellular numbers, but only the WT strain triggers the release of LDH above background levels, suggesting that the WT strain is killing cells while the mutant is accumulating intracellularly. The delay in cytosolic escape observed with the *fptB* mutant strain has not been reported previously for Francisella. Extensive work by multiple laboratories has revealed the process by which Francisella escapes the phagosome early in the replication cycle, which host and bacterial genes are key to this process, and the consequences of delays at this stage (50–54). However, escape from the host cell at the final stage of the intracellular cycle remains an aspect of the Francisella life cycle that is poorly understood. Current models of escape from macrophages involve type A Francisella replicating to high numbers before triggering cell death mediated through caspase 3 (55), during which the bacteria are presumed to be released from the cell (35, 56, 57). While the escape process appears to be tied to the cell death machinery, the control of the timing of cell death and subsequent escape includes both host and bacterial factors. Exactly which molecular steps define the escape path for F. tularensis remain understudied. It has been demonstrated that Francisella triggers autophagy in mouse embryonic fibroblasts and then associates with the autophagic machinery to harvest this microenvironment for nutrients, especially amino acids (58–61). The involvement of autophagy in the F. tularensis life cycle and release in human cells remains largely unexplored but is an intriguing cell process to explore.

A fundamental key to the virulence of Francisella is its ability to invade and replicate intracellularly in many cell types, especially macrophages, where host responses have been extensively studied, revealing the induction of a potent yet atypical inflammatory response that can ultimately lead to sepsis and hypercytokinemia (62, 63). The avoidance of TLR4, yet the stimulation of TLR2/6, AIM2, and STING, produces an immune response that can be less than effective for host survival (28–30) and highlights the extreme virulence of this bacterium. Infection with the *fpt* mutant strain elicited altered inflammatory cytokine secretion: F. tularensis SchuS4Δ*fptB* infection resulted in a 12-h delay in IL-1β secretion and a significantly reduced TNF-α response across all time points measured. The level of attenuation of SchuS4Δ*fptB* in the C57BL/6J mouse model was >700,000 times higher than that of the WT. This high level of attenuation exhibited by F. tularensis SchuS4Δ*fptB* places it among the most attenuated type A-derived vaccine strains (64) and confirms the importance of intracellular replication and host cell interactions early in infection. Although we have not yet reached the lethal dose for this strain, challenge results suggest that F. tularensis SchuS4Δ*fptB* may be overattenuated. Historically, the prime difficulty of live-attenuated vaccine development has been achieving the optimal balance of safety/attenuation and immunogenicity/protection. The reduced inflammatory response elicited by F. tularensis SchuS4Δ*fptB* in the early stages of macrophage infection compared to the one elicited by the WT may indicate that an altered host cell interaction at the time of initial macrophage infection does not guarantee the onset of a long-term protective response.

These data represent the first utilization of an MFS transporter, an amino acid transporter specifically, as an attenuating target in the virulent F. tularensis type A background. Previous efforts were centered upon biosynthetic (16, 18, 65), capsule (19, 66, 67), and virulence (68) genes, but transporters represent a largely untapped source of potentially attenuating targets. Importantly, nutrient transporters, including those of the MFS, may be of increased importance to intracellular pathogens. It is appreciated that bacteria residing in the intracellular space undergo genome reductions, resulting in the shedding of genes involved in transport and metabolism at a rate that is much higher than that of genes involved in replication and defense (69). This implies that any remaining transporter and metabolite genes are necessary for the survival and, possibly, the virulence of the organism. The type A strain of Francisella tularensis is predicted to have 35 MFS transporters and 182 transporters in total (70), representing close to 12% of the SchuS4 protein-coding sequences. This represents a very large pool of potential novel targets for not just vaccines but also antibiotic therapies. Intriguingly, similar gene proportions exist in many clinically relevant pathogenic organisms.

F. tularensis SchuS4Δ*fptB* is the first MFS-based live-attenuated strain in the type A background to demonstrate protection against WT type A Francisella challenge. While the efficacy was relatively modest following vaccination with this strain, these data support the idea of targeting nutrient transporters as part of an attenuation strategy. These strains represent insightful tools allowing more intimate study of the intracellular life cycle of F. tularensis, especially the understudied molecular steps leading to cellular escape.

## MATERIALS AND METHODS

### Bacteria and growth conditions.

Bacterial strains utilized in this study are listed in Table S1 in the supplemental material. Francisella tularensis SchuS4 (BEI, Manassas, VA) was preserved at −80°C in Mueller-Hinton broth (MHB) (BD Microbiology Systems, Sparks, MD) with 15% glycerol added. Complete MHB includes 1% IsoVitaleX (BD, Cockeysville, MD), 0.1% glucose, and 0.25% ferric pyrophosphate and was used for all liquid cultures. Mueller-Hinton agar (MHA) (BD Microbiology Systems, Sparks, MD) was used for solid cultures and was augmented as defined above but also contained 10% defibrinated sheep blood (Lampire Biological Laboratories, Pipersville, PA). When selection for electroporants was being undertaken, kanamycin (Km) was added to MHA to a final concentration of 10 μg/ml. Suicide plasmids used in this study (Table S1) were preserved in Escherichia coli DH5α and grown in Luria-Bertani (LB) broth (BD Microbiology Systems, Sparks, MD) supplemented with 50 μg/ml kanamycin.

### Deletion of F. tularensis genes.

F. tularensis was transformed via electroporation as previously described (13). Briefly, F. tularensis was first grown on MHA at 37°C in 5% CO_2_ for 2 days and then resuspended in 1 ml of 0.5 M sucrose. Bacteria were pelleted and washed three times in 0.5 M sucrose before being resuspended in a final volume of 300 μl. One hundred fifty microliters of a suicide plasmid (13) prepared from 500-ml LB broth cultures (utilizing a Qiagen [Germantown, MD] Midi-Prep kit) was added to this mixture. Electroporation was performed at 1.75 kV, 25 μF, and 600 Ω. After electroporation, cells were allowed to recover in MHB for 2 h at 37°C in 5% CO_2_ and then plated onto kanamycin-containing MHA plates to select for cointegrants. Cointegration of the plasmid was confirmed by using PCR. Plasmid curing was accomplished by growth in MHB containing 10% sucrose. These cultures were plated onto MHA supplemented with 8% sucrose, and colonies arising here were screened by PCR to confirm the loss of the targeted gene and plasmid. Primers utilized for screening can be found in Table S2 in the supplemental material.

### Growth curves.

Growth curves comparing WT F. tularensis and *fpt* mutant strains were performed by using Chamberlain's defined medium (CDM) (Teknova, Hollister, CA) or CDM supplemented with 3 mM isoleucine. Bacteria were grown on MHA plates and resuspended to a starting optical density at 600 nm (OD_600_) of 0.l in liquid medium. Cultures were incubated with shaking at 37°C in 5% CO_2_ for 7.5 h, with OD_600_ readings being taken every 1.5 h.

### Intracellular survival assays.

The ability of *fpt* mutant strains to survive and replicate intracellularly was evaluated in the human THP-1 macrophage cell line (ATCC, Manassas, VA), the human HepG2 hepatic carcinoma cell line (ATCC, Manassas, VA), hMDMs, and BMDMs from C57BL/6J mice.

THP-1 cells were cultivated in RPMI 1640 (Cellgro, Manassas, VA) supplemented with 10% fetal bovine serum (FBS) (Sigma, St. Louis, MO) and 0.1% 1,000× 2-mercaptoethanol (Gibco, Gaithersburg, MD) and maintained at 37°C in 5% CO_2_. Three days prior to the beginning of an assay, THP-1 cells were differentiated by using phorbol myristate acetate (PMA) (Sigma-Aldrich) at a concentration of 50 ng/ml for 24 h. Medium was then changed to supplemented RPMI 1640 as described above. For assays to assess bacterial replication, cells were infected with either F. tularensis SchuS4 or *fpt* mutants at a multiplicity of infection (MOI) of 100 for 2 h. Following the 2-h infection, cells were washed twice with phosphate-buffered saline (PBS) and incubated in RPMI 1640 containing 50 μg/ml gentamicin (Gibco, Gaithersburg, MD) for 1 h. At 3 hpi, cells were washed again twice with PBS and placed into fresh RPMI 1640 lacking gentamicin. Intracellular bacterial replication was assayed at 3, 24, and 48 h postinfection by lysing cells with a solution containing 0.02% SDS in PBS, followed by serial dilution and plating of the bacteria onto MHA. Culture supernatants were saved for downstream analyses. Bacterial doublings were calculated by utilizing the formula [log_10_ *T_n_* − log_10_ *T*_(*n* − 1)_] × 3.32, where *T_n_* is the number of CFU at one time point and *T*_(*n* − 1)_ is the number of CFU at the prior time point. For assays to assess bacterial escape kinetics, the infection and washing steps were repeated as described above through 3 hpi. Gentamicin-containing medium was not washed off cells, and thus, cells were left in 50 μg/ml gentamicin for the duration of the assay. CFU were sampled as described above at the time courses indicated in the figure legends.

To assess the appearance of bacteria in the supernatants, THP-1 cells were infected and washed as described above. Gentamicin-containing medium was removed at 3 hpi, and cells were placed into antibiotic-free medium until the enumeration of CFU at 3, 9, 15, and 21 hpi. Supernatants were removed from the wells at the times specified above, serially diluted, and plated onto MHA. Intracellular bacteria were then enumerated as described above.

HepG2 cells were cultivated in minimal essential medium (MEM) supplemented with 10% FBS and maintained at 37°C in 5% CO_2_. To assess the intracellular survival abilities of *fpt* mutant strains compared to that of WT F. tularensis, HepG2 cells were also seeded at a density of 1 × 10^6^ cells per well in 12-well plates and infected with either F. tularensis SchuS4 or *fpt* mutant strains at an MOI of 300 for 2 h. After the 2-h infection period, cells were washed twice with PBS and then incubated in MEM containing 50 μg/ml gentamicin for 1 h. Next, cells were either left in gentamicin-containing medium for the duration of the experiment or washed twice with PBS and then incubated in antibiotic-free MEM for the duration of the experiment. Bacterial replication was assayed at 3, 24, and 48 h postinfection by lysing the cells with 0.02% SDS–PBS and plating the bacteria onto MHA.

### Supplemented intracellular survival assays.

The ability of isoleucine supplementation to rescue the intracellular replication of F. tularensis SchuS4Δ*fptB* was examined in THP-1 macrophage-like cells. THP-1 cells were prepared and infected as described above. Immediately before the start of the assay, all cells were washed and placed into either standard RPMI 1640 or RPMI 1640 supplemented with 3 mM isoleucine. Such medium conditions were maintained throughout the entirety of the assay before CFU enumeration at 24 h postinfection.

### Isolation and culture of mouse bone marrow-derived macrophages.

Femurs were extracted from 6- to 8-week-old C57BL/6J mice (University of Maryland, Baltimore [UMB], Veterinary Resources Breeding Facility) and flushed with Dulbecco's minimal essential medium (DMEM) supplemented with 10% low-endotoxin FBS (Gemini Bioproducts, West Sacramento, CA), 30% L929 cell supernatants (as a source of colony stimulating factor 1 [CSF-1]), 1% nonessential amino acids (ThermoFisher Scientific, Waltham, MA), 1% HEPES (ThermoFisher Scientific, Waltham, MA), and 1% penicillin-streptomycin (ThermoFisher Scientific, Waltham, MA). Bone marrow cells were passed through a 70-μm nylon mesh (ThermoFisher Scientific, Waltham, MA) to remove debris and placed into a T175 flask (Costar, Corning, NY) for culture and differentiation. Fresh medium was added on the day after extraction and every other day subsequently for at least 1 week. The day before an assay, cells were scraped, spun down at 1,100 rpm (125 × *g*) for 10 min, and resuspended at a concentration of 5 × 10^5^ cells per ml in medium lacking penicillin-streptomycin. Cells were plated at a concentration of 5 × 10^5^ cells per well in a 24-well plate (Costar, Corning, NY).

### Isolation and culture of human monocyte-derived macrophages.

Monocyte-derived macrophages (MDMs) were isolated from 100 ml whole human blood gathered in EDTA tubes (BD Microbiology Systems, Sparks, MD) by the University of Maryland, Baltimore, Center for Vaccine Development clinical staff. Blood was diluted 1:2 in PBS before being added to 15 ml of Ficoll (GE Healthcare, Laurel, MD) in SepMate tubes (Stem Cell Technologies, Cambridge, MA). SepMate tubes were spun at 1,200 × *g* for 15 min at room temperature. Red blood cells were depleted by using ammonium chloride-potassium (ACK) lysis buffer (Gibco, Gaithersburg, MD). Isolated MDMs were cultured for 1 week before the start of an assay in T75 flasks (Costar, Corning, NY) with RPMI 1640 supplemented with 10% low-endotoxin FBS (Gemini Scientific, West Sacramento, CA), 1% nonessential amino acids (ThermoFisher Scientific, Waltham, MA), 1% HEPES (ThermoFisher Scientific, Waltham, MA), and 1% sodium bicarbonate (ThermoFisher Scientific, Waltham, MA). Medium was changed daily. Two days before the start of an assay, cells were scraped from the flask, enumerated, and plated at a density of 5 × 10^5^ cells per well in a 24-well plate (Costar, Corning, NY). These studies were approved by the University of Maryland, Baltimore, Institutional Review Board.

### Complementation of mutants.

A plasmid containing *fptB* under the control of the *F. tularensis guaB* promoter, named p*FT906-fptB* (13), was electroporated into the F. tularensis SchuS4 mutant strain as described above. The complemented mutant was then tested via an invasion assay in THP-1 macrophage-like cells for reversion to WT growth mechanics.

### Intracellular replication time course assay.

The timing of host cellular escape of *fpt* mutant strains compared to that of WT F. tularensis SchuS4 was evaluated in THP-1 macrophage-like cells. Differentiated THP-1 cells were seeded at a density of 1 × 10^6^ cells and then infected with either F. tularensis SchuS4 or the *fpt* mutant strain at an MOI of 100 for 2 h. Following the 2-h infection, cells were washed twice with PBS and then incubated in RPMI 1640 containing 50 μg/ml gentamicin for the remainder of the assay. Supernatants were collected for subsequent measurement of levels of LDH and cytokine release, and CFU were determined by lysing the cells with a 0.02% SDS solution in PBS, followed by serial dilution and plating of the bacteria onto MHA at 3, 9, 15, 21, 27, and 33 h postinfection.

### Lactate dehydrogenase release assay.

LDH release was measured by using a CytoTox 96 kit (Promega, Madison, WI) according to the manufacturer's protocol. Measurement of samples was done by using a VersaMax plate reader (Molecular Devices, Sunnyvale, CA) at a 490-nm wavelength.

### Cytokine ELISAs.

To assay the secretion of IL-1β and TNF-α from THP-1 cells, enzyme-linked immunosorbent assay (ELISA) kits from R&D Systems (Minneapolis, MN) were used. Supernatants collected at 3, 9, 15, 21, 27, and 33 h postinfection were assayed according to the manufacturer's protocol and read by using a VersaMax plate reader at 450 nm, with correction set at 540 nm.

### Mouse survival studies.

Six- to eight-week-old male and female C57BL/6J mice were housed in the University of Maryland animal biohazard safety level 3 (ABSL-3) facility for the duration of the studies. All experiments were performed according to protocols approved by the UMB Institutional Animal Care and Use Committee (IACUC). To ascertain whether the *fpt* mutant strain was attenuated, a mouse infection model was used. Groups of 4 C57BL/6J mice per dosage concentration (2 males and 2 females) were anesthetized and inoculated intranasally with either WT F. tularensis SchuS4 or the *fpt* mutant suspended in 20 μl of PBS. Mice were monitored daily for survival and clinical signs of infection (weight loss, lethargy, and ruffling of fur) for 14 days postinfection.

Mouse health was scored based on the following criteria: condition 1 for normal activity, where mice run freely and energetically around the cage and resist when picked up; condition 2 for mice that are slower than usual and offer less resistance when picked up; condition 3 for mice that exhibit a hunched posture, move very slowly, and display ruffled, dull fur and squinted eyes; and condition 4 for mice that are almost entirely sedentary, hunched, and either unresponsive or barely responsive to prodding and that have fur that is very ruffled and dull. Mice reaching a clinical score of 4 or losing >20% of their body weight were euthanized as required by the UMB IACUC.

### Mouse challenge studies.

Six- to eight-week-old male and female C57BL/6J mice were immunized as described above by using a single vaccination regimen. Mice received ∼1 × 10^6^ CFU of F. tularensis SchuS4Δ*fptB* and were challenged with WT F. tularensis SchuS4 intranasally 4 weeks later with the doses outlined in Table 2. After challenge, all mice were monitored daily to check for clinical signs and weight loss as described above. The delay in the time to death for vaccinated, challenged mice was calculated by using the average day of death postchallenge for the unvaccinated control group and the average day of death for vaccinated mice that succumbed to challenge. The average day of death for each group was calculated, and the average day of death for the control group was subtracted, giving the delay in the time to death per group. Mice that survived challenge were excluded from these calculations.

### Statistical analysis.

By using GraphPad Prism 7.0 (GraphPad Software, San Diego, CA), two-way analysis of variance (ANOVA) with Tukey's posttest was performed to assess statistical significance between log-transformed bacterial CFU counts, LDH release levels, and cytokine ELISA data (*P* < 0.05). A two-sided *t* test was utilized to determine significance between calculated bacterial doubling values (*P* < 0.05). Calculated *P* values for the *t* tests were adjusted by using the Benjamini, Krieger, and Yekutieli method with a false discovery rate of 1%. In all instances, WT F. tularensis SchuS4 served as the reference strain for all statistical tests.

## Supplementary Material

Supplemental material
