# Supplementary material for: Deletion of the Major Facilitator Superfamily Transporter fptB Alters Host Cell Interactions and Attenuates Virulence of Type A Francisella tularensis
Source: Infect Immun. 2018 Feb 20;86(3):e00832-17. doi: 10.1128/IAI.00832-17 (PMC5820938; doi:10.1128/IAI.00832-17)
Supplement: Supplemental material [file supp_86_3_e00832-17__index.html]

Supplemental material 

# Deletion of the Major Facilitator Superfamily Transporter *fptB* Alters Host Cell Interactions and Attenuates Virulence of Type A Francisella tularensis

## Supplemental material

- Supplemental file 1 -

  Fig. S1. Deletion and purification of an *F. tularensis* SchuS4 strain lacking the *fptB* gene. Fig. S2. Complementation of the *fptB* gene restores wild-type growth kinetics in THP-1 cells. Table S1. Plasmids and strains utilized in this study. Table S2. Primers utilized in this study.

  PDF, 204K
