# Supplementary material for: Deletion of the Major Facilitator Superfamily Transporter fptB Alters Host Cell Interactions and Attenuates Virulence of Type A Francisella tularensis
Source: Infect Immun. 2018 Feb 20;86(3):e00832-17. doi: 10.1128/IAI.00832-17 (PMC5820938; doi:10.1128/IAI.00832-17)
Supplement: Supplemental material [file IAI.00832-17_zii999092339s1.pdf]

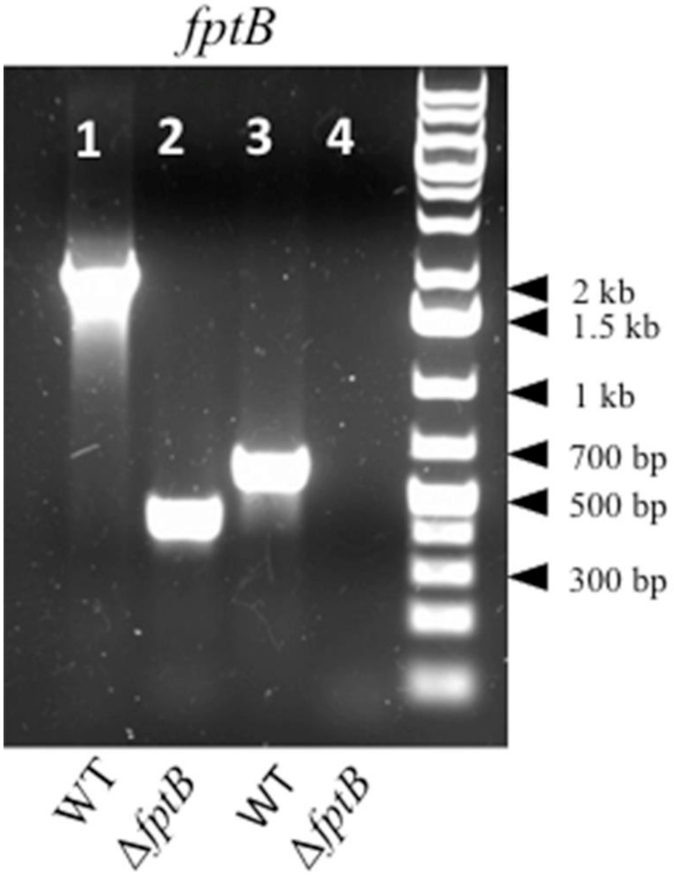

Figure S1: Deletion and purification of an *F. tularensis* SchuS4 strain lacking the *fptB* gene. DNA was isolated and the presence or absence of the *fptB* gene was ascertained via PCR. Lanes 1 and 2 represent the gene-specific PCR product amplified with primers located in the upstream and downstream flanks in either WT *F. tularensis*, or the strain lacking *fptB.* Lanes 3 and 4 represent the PCR product from primers located within the *fpt* gene.

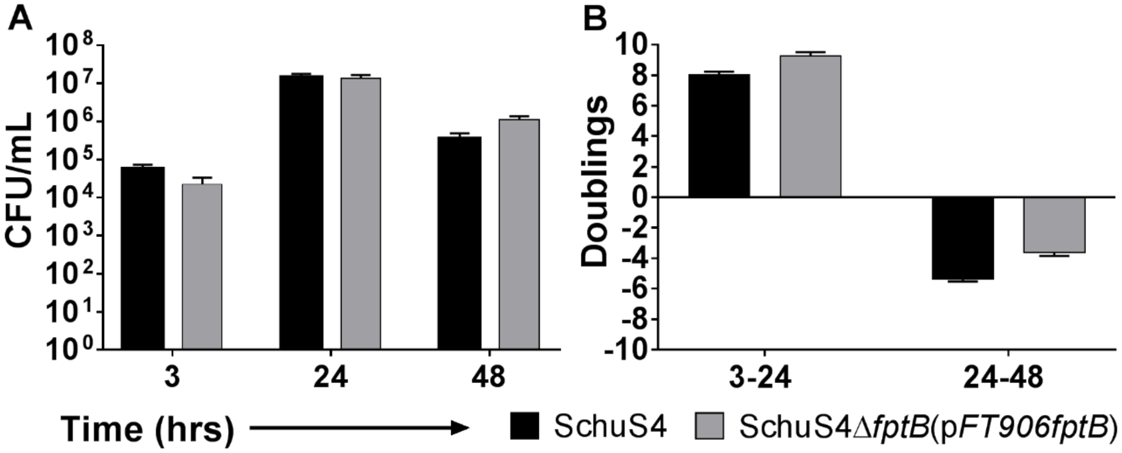

Figure S2: Complementation of the *fptB* gene restores wildtype growth kinetics in THP-1 cells. THP-1 cells were infected at an MOI of 100 for 2 hours with *F. tularensis* WT or SchuS4Δ*fptB*. Cells were then washed twice with PBS and incubated with 50 µg/mL gentamicin for 1 hour before being returned to media lacking gentamicin Intracellular bacteria were enumerated at 3, 24, and 48 hours post-infection. Data are presented as mean $\pm$ SEM with three biological replicates for each experiment. Data are of a single representative experiment (n = 2). (Average CFU analyzed by two-way ANOVA and calculated doublings analyzed by two-sided t test with Benjamini, Krieger, and Yekutieli *p* value adjustment).

Table S1: Plasmids and strains utilized in this study

Strain	Characteristics	Source
Francisella tularensis SchuS4	Wildtype	BEI
SchuS4Δ*fptB*	SchuS4 deletion of *FTT_0056c*	This study
SchuS4Δ*fptG*	SchuS4 deletion of *FTT_1291*	This study
SchuS4Δ*fptB*(pFT906*fptB*)	Trans-complemented *fptB* mutant strain	This study
SchuS4Δ*fptG*(pFT906*fptG*)	Trans-complemented *fptG* mutant strain	This study
**Plasmid**	**Characteristics**	**Source**
pFT893ΔfptB	Suicide plasmid for *FTT_0056c*	(1)
pFT893ΔfptG	Suicide plasmid for *FTT_1291*	(1)
pFT906*fptG*	Trans-complement plasmid of *fptG*	(1)
pFT906*fptB*	Trans-complement plasmid of *fptB*	(1)

Table S2: Primers utilized in this study

Primer	Sequence	Function
SchuS4_inB_F	TTTGCCTTTGCCGCAAGCTTTC	Confirm deletion of *fptB*
SchuS4_inB_R	CCAATAATTGGTGAGCCGATAGCC	Confirm deletion of *fptB*
SchuS4_inG_F	CCGCTTTCATGTGGTCATATGC	Confirm deletion of *fptG*
Schus4_inG_R	GCAACTAATGCCCATGTTGGAG	Confirm deletion of *fptG*
Fpt_G Forward	TCTAGGTGGTGTCACCAAAC	External primers flanking *fptG*
Fpt_G Reverse	AGCAAACCGCTATGGTATCC	External primers flanking *fptG*
Fpt_B_SchuS4_F	CCCTGGACTGAACTGCTTAT	External primers flanking *fptB*
Fpt_B_SchuS4_R	CGGTTAGGCATCTACAAGGA	External primers flanking *fptB*
plasmid amplifier_F	GCTGCAAGGCGATTAAGTTGGGTAACG	Confirm plasmid cointegration
plasmid amplifier_R	ACTGTCGCAAACTATCACGGCTACCAC	Confirm plasmid cointegration

References cited in supplemental figures and tables

1. Marohn ME, Santiago AE, Shirey KA, Lipsky M, Vogel SN, Barry EM. 2012. Members of the Francisella tularensis Phagosomal Transporter Subfamily of Major Facilitator Superfamily Transporters Are Critical for Pathogenesis. Infection and Immunity 80:2390-2401.
